# Rectal endometriosis presenting as toxic megacolon

**DOI:** 10.4322/acr.2021.319

**Published:** 2021-09-03

**Authors:** Luis Enrique Rosario Alvarado, Hisham Bahmad, Odille Mejia, Heather Hollembeak, Robert Poppiti, Lydia Howard, Kiranmayi Muddasani

**Affiliations:** 1 Mount Sinai Medical Center, Department of General Surgery, Miami Beach, FL, USA; 2 Mount Sinai Medical Center, The Arkadi M. Rywlin M.D. Department of Pathology and Laboratory Medicine, Miami Beach, FL, USA; 3 Florida International University, Herbert Wertheim College of Medicine, Miami, FL, USA

**Keywords:** Case Reports, Megacolon, Toxic, Endometriosis, Colon

## Abstract

**Background:**

The bowel is the most common site of extragenital endometriosis, with involvement of the locoregional sigmoid colon and anterior rectum seen most often. The clinical presentation varies depending on how soon patients seek medical care, thus requiring changes in management strategies. Endometriosis can cause a life-threatening surgical emergency with progressive obliteration of the bowel lumen leading to obstruction and late complications including toxic megacolon and transmural necrosis.

**Case presentation:**

We report the case of a 41-year-old woman presenting with an acute abdomen and complete large bowel obstruction complicated by sepsis and toxic megacolon. The patient underwent emergency total colectomy with ileostomy. Medical history was significant for chronic, vague, and episodic lower abdominal pain self-medicated with herbal tea and laxatives. Pathologic examination demonstrated colonic endometriosis within the bowel wall as the cause of obstruction, ischemia, and transmural necrosis.

**Conclusions:**

Although a rare clinical entity, this case highlights two important points. First, it demonstrates the value of performing proper and complete clinical work up to rule out or in all possible causes of colonic obstruction, including intestinal endometriosis. Second, it suggests a potential benefit of a formalized multidisciplinary approach, including surgery, in the management of medically unresponsive endometriosis. In conclusion, this case shows that endometriosis can cause life-threatening colonic obstruction in women of childbearing age. Prompt early intervention is warranted, particularly when obstruction is only partial and ischemia has not supervened, to conceivably prevent the development of a toxic megacolon requiring colectomy and avoid late complications.

## INTRODUCTION

Endometriosis is defined as "the presence of endometrial glands and stroma outside the uterine cavity."^1^ Extragenital endometriosis can be subdivided into pelvic or extrapelvic endometriosis.^2^ While pelvic endometriosis mostly involves the uterosacral ligaments (70%) and the vagina (14%),^3^ the most common site for extrapelvic endometriosis is the gastrointestinal tract,^4^ particularly the rectum and sigmoid colon (up to 95%).^5^^,^^6^ Gastrointestinal endometriosis is estimated to affect 3.8-37% of endometriosis patients.^7^ Patients with intestinal endometriosis may remain asymptomatic or present with cyclic non-specific abdominal pain, nausea, vomiting, diarrhea, and/or constipation among other symptoms.^8^ Intestinal obstruction is an extremely rare presentation with reported incidence between 0.1–0.7% of all endometrioses.^9^^,^^10^

Pathologically, intestinal endometriosis can manifest as "deeply implanted foci" within the mucosa or muscularis, or superficially infiltrating the bowel serosa or subserosa.^11^ "Deeply implanted foci" of intestinal endometriosis may be microscopic, or a large infiltrating mass involving the entire bowel wall with narrowing of the lumen and obstruction.^12^^,^^13^ Obliteration of the lumen can lead not only to obstruction, but also ischemia, necrosis, and sepsis. Importantly, the differential diagnosis of colorectal endometriosis includes carcinoma of the colon and rectum, which can be difficult given similar colonoscopic and radiologic findings.^14^

We report a rare case of rectal endometriosis presenting as toxic megacolon in a 41-year-old woman. The patient did not have any previous symptoms suggesting endometriosis, and, on presentation, urgent surgery was required. Pathological examination yielded the diagnosis of intestinal endometriosis with "deeply implanted foci" within the muscularis propria. Only rare cases of endometriosis confined to the rectum without pelvic involvement were noted in the literature.

## CASE REPORT

A 41-year-old woman with no significant medical or social history presented to our emergency department with acute, non-radiating, diffuse, sharp abdominal pain, and abdominal distention. The symptoms started 5 days earlier, and had been getting worse with time. Onset was gradual and constant in nature, moderate in severity, and was associated with fatigue, diarrhea, as well as nausea, and vomiting. The patient self-treated with herbal tea and laxatives with no improvement. She denied fever or chills, dizziness, lightheadedness, bloody stools, or melena. The patient also denied any gynecological symptoms or history of gynecological/obstetric surgeries.

On physical examination, she appeared ill. Her blood pressure was 102/50 mmHg; she was tachycardic (pulse was 118 beats per minute); the oral temperature was 36.8^o^c and her respiratory rate was 20. Her BMI was 24.89 kg/m^2^. The patient's abdomen was distended, tympanitic, and diffusely tender with rebound and guarding, and her skin was pale, raising suspicion of peritonitis. There were no palpable masses or rigidity.

Laboratory studies were done in the emergency department showing leukocytosis with a white blood cell (WBC) count of 28.56 x 10^3^/μL (reference range 4.8 - 10.8 x 10^3^/μL) and neutrophilia with left shift (87% segmented neutrophils (reference range 42 - 75%) and 6% neutrophil bands (reference range 0 - 10%)). The absolute neutrophil count was 26.56 x 10^3^/μL (reference range 1.8 - 7.2 x 10^3^/μL), and lymphocytes and monocytes were decreased. Red blood cell (RBC) count was 5.98 x 10^6^/μL (reference range 3.93 - 5.22 x 10^6^/μL), hemoglobin 17.3 g/dL (reference range 12.0 - 16.0 g/dL), and hematocrit 54.2% (reference range 37.0 - 47.0%). Red cell indices were within normal limits. Electrolyte levels were as follow: sodium 135 mMol/L (reference range 136 - 145 mMol/L), potassium 4.1 mMol/L (reference range 3.5 - 5.1 mMol/L), chloride 97 mMol/L (reference range 98 - 107 mMol/L), calcium 8.9 mg/dL (reference range 8.5 - 10.1 mg/dL), anion gap 19.9 mMol/L (reference range 10 - 20 mMol/L), and CO_2_ 22.2 mMol/L (reference range 21.0 - 32.0 mMol/L). Hepatic profile was as follows: ALT/SGPT 42.0 U/L (reference range 13 - 56 U/L), AST/SGOT 35.0 U/L (reference range 15 - 37 U/L), total protein 8.3 g/dL (reference range 6.4 - 8.2 g/dL), and total bilirubin 0.55 mg/dL (reference range 0.20 - 1.00 mg/dL). The patient had high blood creatinine level at 1.37 mg/dL (reference range 0.55 - 1.02 mg/dL) and high blood urea nitrogen (BUN) level at 35.0 mg/dL (reference range 7 - 18 mg/dL). Her alkaline phosphatase level was slightly elevated at 133 U/L (reference range 46 - 116 U/L) and lactate level was markedly elevated at 11.8 mMol/L (reference range 0.4 - 2.0 mMol/L).

**Figure 1:**
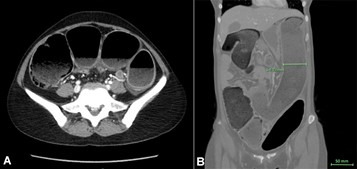
Abdominal computerized tomography (CT) scan with intravenous contrast. Results **A** and **B** showed extensive colonic intramural air (pneumatosis intestinalis) prominently involving the ascending colon with significant and potentially life-threatening severe dilation of the entire colon, maximally measuring 6.78 cm in diameter without evidence of free air.

A computerized tomography (CT) scan with intravenous contrast was performed, showing extensive colonic intramural air (pneumatosis intestinalis) prominently involving the ascending colon with significant and potentially life-threatening severe dilation of the entire colon, maximally measuring 6.78 cm in diameter. There was no evidence of free air (Figure 1A, B). The large bowel was tortuous and dilated with extensive air-fluid levels and a small amount of free fluid in the pelvis (Figure 1A, B).

Due to the life-threatening condition and the pre-operative provisional diagnosis of toxic megacolon with colitis, she was taken immediately to the operating suite for exploratory laparotomy. Upon the initial midline incision to access the abdomen, a markedly distended colon extending from the rectum to the cecum was immediately noticed. No sign of bowel perforation was appreciated after inspecting the bowel from the ligament of Treitz distally However, it was noted that the rectum had a long area of stricture along the level of the peritoneal reflection. Simultaneous intra-operative colonoscopy was performed, and it showed no obvious intraluminal mass. A total abdominal proctocolectomy was then performed. The abdomen was irrigated and closed to finally proceed with an end ileostomy creation. The patient was transferred to the intensive care unit (ICU).

**Figure 2:**
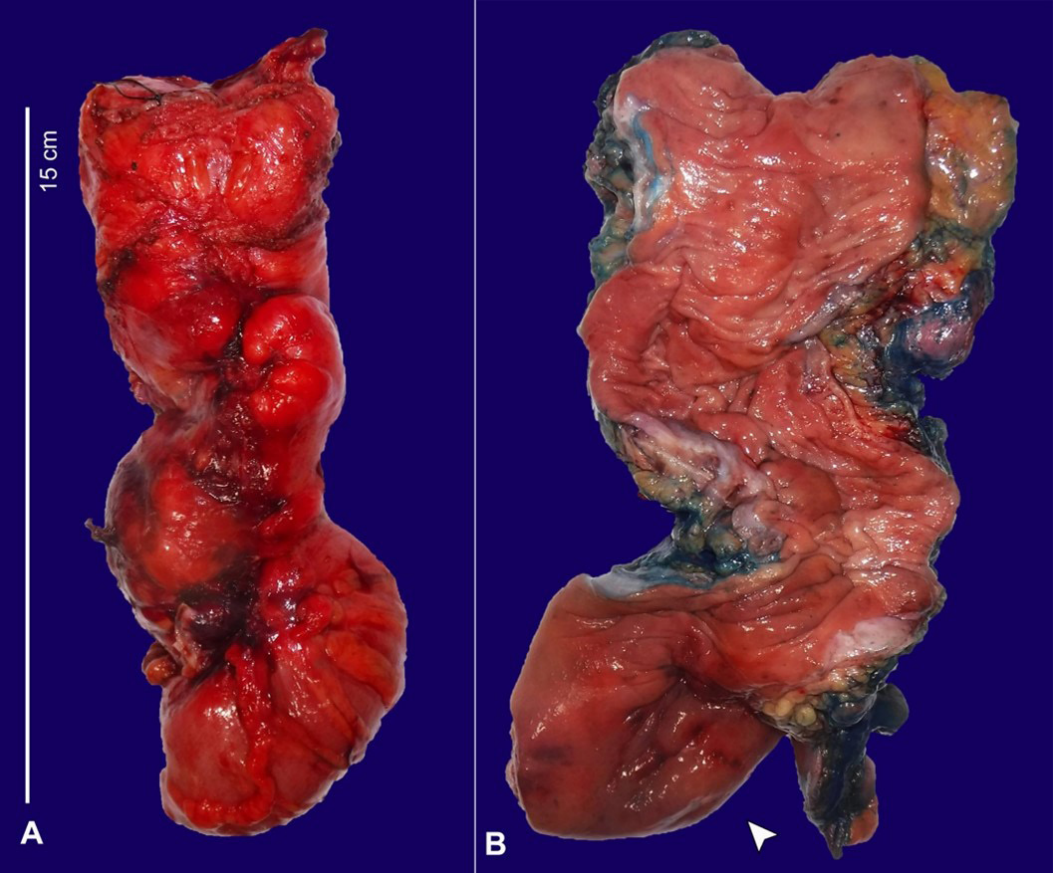
Gross examination of the resected colon. **A** – Segment of rectum measuring 15 cm in length and approximately 4.5 cm in average diameter with mucosa demonstrating diffuse necrosis; **B** – Area of dilatation identified at the distal end of the resected rectum measuring up to 7 cm in average diameter (arrowhead).

**Figure 3:**
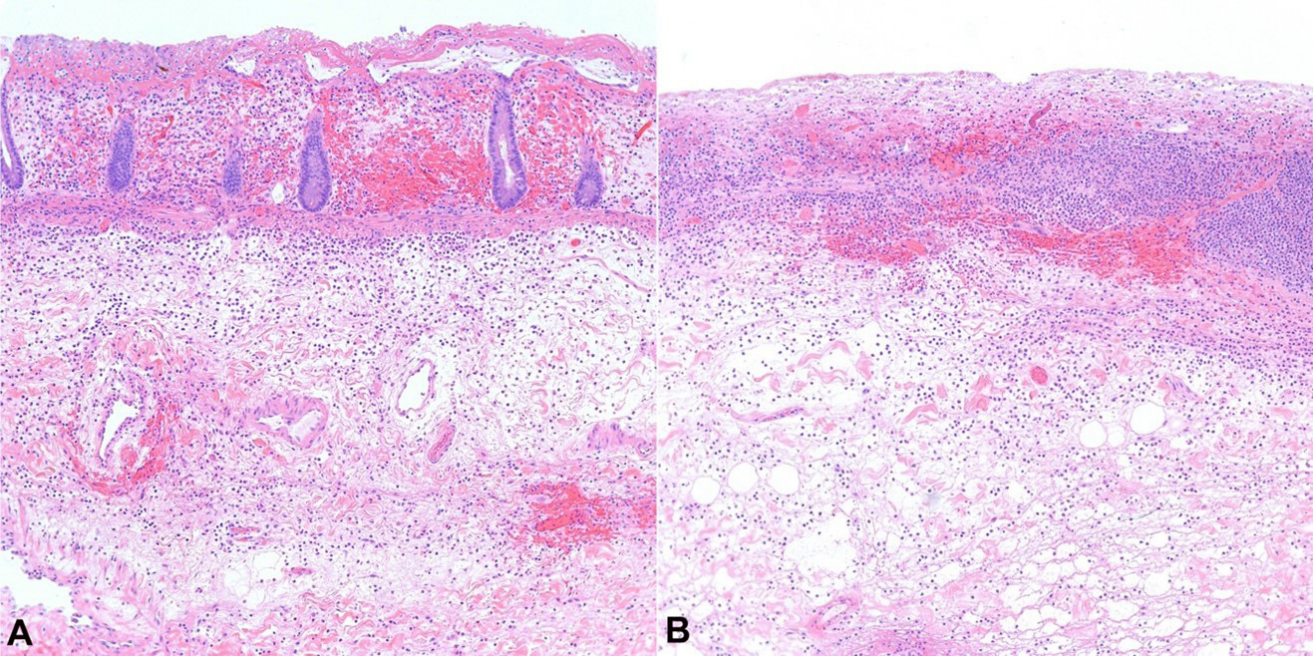
Microscopic examination showing areas of extensive necrosis (H&E stain). **A** – Histopathologic examination of the resected colon demonstrated extensive, nearly transmural necrosis; **B** – with moderate to severe acute and chronic inflammation. Microscopic images were examined at 100x.

Two resected segments of the colon were received by pathology from surgery. One segment measured 91 cm in length and approximately 7 cm in average diameter, and the other, a segment of the rectum, measured 15 cm in length and approximately 4.5 cm in average diameter. Opening both segments longitudinally revealed diffusely necrotic mucosa (Figure 2A), which was seen microscopically as extensive, nearly transmural necrosis (Figure 3A) with moderate to severe acute and chronic inflammation (Figure 3B). There was also an area of dilatation identified at the distal end of the resected rectum measuring up to 7 cm in average diameter (Figure 2B).

**Figure 4:**
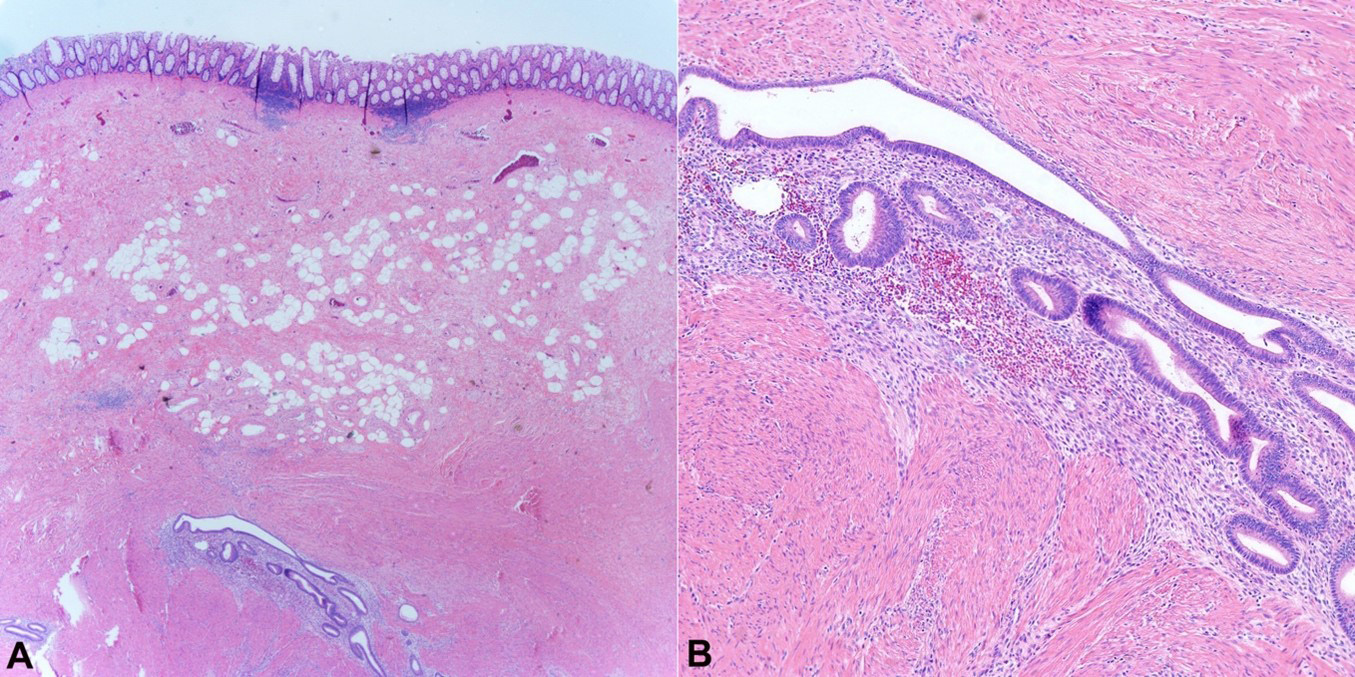
Microscopic examination showing areas of intestinal endometriosis (H&E stain). Histopathologic examination demonstrating colonic mucosa with endometrial glandular epithelium (black arrow) and endometrial stroma (red arrow) within the submucosa and muscularis propria. Microscopic images were examined at **A** – 25x; and **B** – 100x, respectively.

This represented an area of extensive endometriosis with associated fibrosis and foci of mucosal necrosis (Figure 4). Results were discussed with the patient and referral to a gynecologist for continued monitoring of her endometriosis was recommended.

The patient had an extended postoperative course with the development of an ileus on postoperative day (POD) five. Total parenteral nutrition (TPN) was started on POD six due to the concern for an extended course of obstruction/ileus. She remained on antibiotic coverage with Ciprofloxacin and Metronidazole for seven days that was discontinued when the WBC count normalized. She experienced the return of bowel function on POD ten but with persistently high nasogastric tube outputs.

On POD fourteen, there was no return of bowel function, and due to concerns of kinking or stenosis, the patient underwent an ileoscopy. The procedure showed a mildly inflamed peri-anastomosis ileum with no other significant findings. At the end of her hospital course on POD nineteen, there was complete resolution of her initial symptoms, and the ileostomy was functioning with no signs of dumping or dehydration. The patient was discharged home after reviewing all instructions, including ileostomy maintenance and care. She reported no issues, and successfully returned to daily activities at her 2-week post-op appointment.

## DISCUSSION

Endometriosis is defined as "the presence of endometrial glands and stroma outside the uterine cavity."^1^ It predominantly occurs in the ovaries, followed by soft tissues (abdominal wall, uterine cervix and vagina, inguinal, vulva, etc.), gastrointestinal tract, and urinary tract among others. In a study by Lee et al*.* 15 including 1,350 women, gastrointestinal endometriosis accounted for 0.3% of cases. It has been proposed that multiple factors might contribute to the pathogenesis of this clinical entity.^16^ However, the most widely accepted theory is Sampson’s theory which was introduced in 1927. It stipulates that viable endometrial cells reflux backward with the blood flow in rare cases of retrograde menstruation and get implanted in extragenital locations then grow to form peritoneal lesions.^17^ Celomic metaplasia is another theory that describes the ability of normal cell derivatives of the primitive parietal peritoneum to transform into endometrial tissue.^18^ Other theories include the presence of cells of Mullerian origin within the peritoneal cavity that are triggered by certain cellular and genetic factors to form endometrial tissue (embryonic rest theory),^19^ possible transport of endometrial tissues through the vasculature and lymph nodes to implant in nearby organs, particularly the ovaries and gastrointestinal organs (lymphatic and vascular metastasis theory) which best explains intestinal endometriosis as in our case,^20^ repetitive microtraumatizations during reproductive life leading to tissue injury and repair and eventually prompting the implantation of endometrial cells at abnormal locations (TIAR) including implantation endometriosis post-caesarean section,^21^ nerve injuries,^22^ and implantation of stem/progenitor cells from the shedding endometrium due to retrograde neonatal uterine bleeding.^23^

The sigmoid and rectum are the most common sites of bowel involvement followed in descending order by the appendix, the terminal ileum, the cecum, and other parts of the large and small intestine, including Meckel diverticulum.^4^ Intestinal involvement is confined to the serosa or subserosa and is hence asymptomatic or subclinical. If the rectosigmoid is affected, as in our case, it is usually involved by a solitary lesion several centimeters in length, in contrast to ileal involvement, where it is usually multifocal.^24^

Grossly, the bowel appears indurated and often angulated by an ill–defined mass, usually non-circumferential. The serosa may be puckered and covered by adhesions.^25^ Sectioning shows a firm, gray–white solid mass due to the underlying thickened colonic wall muscle layer. Nevertheless, the overlying mucosa remains intact unless complicated by an obstruction or ischemic changes leading to necrosis, which was evident in our case. Although extremely rare, 1–2.5% of patients with gastrointestinal endometriosis require bowel resection for symptomatic disease secondary to complications.^26^

Multiple case reports emphasize the challenging difficulty of diagnosing endometrial disease when it presents in the intestines.^27^ The symptoms of intestinal endometriosis vary according to the site of involvement and dependence on the menstrual cycle, which are both of diagnostic importance. Rectosigmoid endometriosis can cause crampy abdominal pain, alterations in bowel habits, pressure on the coccyx, perimenstrual flatulence, bleeding, as well as rare ileus phenomenon.^28^ Both intestinal endometriosis and colorectal cancer may present with similar symptoms such as rectal bleeding, hence rendering making the diagnosis of endometriosis indeed challenging.^12^^,^^14^

Endometriosis can mimic malignancy on biopsy. Unusual histological types of colonic and rectal adenocarcinomas can be encountered, including cancers from the genital tract such as endometrioid and serous carcinomas which may arise in endometriosis.^29^ Therefore, the differential diagnosis of intestinal endometriosis is wide and includes, besides cancer, inflammatory bowel disease, and ischemia.^26^ In a more concerning scenario, colonic endometriosis may manifest as an acute abdomen resulting from perforation and associated peritonitis.^14^ To the best of our knowledge, only one case has been previously reported in the literature delineating the presentation of intestinal endometriosis as toxic megacolon by Alexandrino *et al.* in 2018.^30^ After the diagnosis of occlusion was assumed, the patient underwent an exploratory laparotomy.^30^ Although in cases of intestinal obstruction, laparoscopic evaluation is recommended leaving the patient with a small periumbilical scar, which is considered a less invasive surgical procedure and is almost always preferable, an open laparotomy with a large incision and potentially far more extensive post-operative adhesions might be inevitable, especially in cases of toxic megacolon as demonstrated in our case. Nevertheless, a history of endometriosis or gynecological symptoms should raise the index of suspicion and help in the diagnosis of intestinal endometriosis.^31^

Both CT colonography and magnetic resonance imaging (MRI) are helpful in detecting rectosigmoid endometriosis, but they lack specificity.^32^ Attention should be given to differentiate the usually symmetric and segmental mural thickening of acute colitis from the asymmetric and rather focal wall thickening of colorectal adenocarcinomas or those found in carcinomatosis or endometriosis.^33^ Despite the fact that endometriosis is not neoplastic, a study by Benoit et al.^34^ demonstrated that up to 1% of patients will develop an endometriosis-associated neoplasm probably due to hormonal factors and genetic anomalies involving loss of heterozygosity on chromosome 5q. Therefore, the key to managing patients with symptomatic intestinal endometriosis refractory to medical therapy is surgical resection of the affected colon, especially in cases of life-threatening complications, such as our case, to alleviate symptoms and prevent potential cancer development. The overall prognosis, however, is excellent.

In conclusion, although very rare, the diagnosis of intestinal endometriosis should always be in the differential as a possible cause of proctocolitis and toxic megacolon. It is difficult to differentiate endometriosis from malignancy based on clinical history or imaging, but pathological examination remains the gold standard for diagnosis.
